# Biosynthesis of soluble carotenoid holoproteins in *Escherichia coli*

**DOI:** 10.1038/srep09085

**Published:** 2015-03-13

**Authors:** Céline Bourcier de Carbon, Adrien Thurotte, Adjélé Wilson, François Perreau, Diana Kirilovsky

**Affiliations:** 1 Commissariat à l'Energie Atomique (CEA), Institut de Biologie et Technologies de Saclay (iBiTec-S), 91191 Gif sur Yvette, France; 2 Centre National de la Recherche Scientifique (CNRS), UMR 8221, 91191 Gif sur Yvette, France; 3 Phycosource, 13 boulevard de l'Hautil, 95092 Cergy Cedex, France; 4 INRA, Institut Jean-Pierre Bourgin, UMR 1318, ERL CNRS 3559, Saclay Plant Sciences, RD10, F-78026 Versailles, France; 5 AgroParisTech, Institut Jean-Pierre Bourgin, UMR 1318, ERL CNRS 3559, Saclay Plant Sciences, RD10, F-78026 Versailles, France

## Abstract

Carotenoids are widely distributed natural pigments that are excellent antioxidants acting in photoprotection. They are typically solubilized in membranes or attached to proteins. In cyanobacteria, the photoactive soluble Orange Carotenoid Protein (OCP) is involved in photoprotective mechanisms as a highly active singlet oxygen and excitation energy quencher. Here we describe a method for producing large amounts of holo-OCP in *E.coli*. The six different genes involved in the synthesis of holo-OCP were introduced into *E. coli* using three different plasmids. The choice of promoters and the order of gene induction were important: the induction of genes involved in carotenoid synthesis must precede the induction of the *ocp* gene in order to obtain holo-OCPs. Active holo-OCPs with primary structures derived from several cyanobacterial strains and containing different carotenoids were isolated. This approach for rapid heterologous synthesis of large quantities of carotenoproteins is a fundamental advance in the production of antioxidants of great interest to the pharmaceutical and cosmetic industries.

The human body is constantly exposed to external (ultraviolet radiation, pollution, cigarette smoke, toxic chemicals) and internal (side reactions of respiration, oxidation of nutrients) factors which induce the formation of Reactive Oxygen Species (ROS). Due to the harmful effects of ROS, the pharmaceutical and cosmetic industries have a significant interest in the production of new antioxidant molecules. For many applications, the medium in which the anti-oxidant effect is desired is water-based and requires a hydrophilic antioxidant. Water soluble carotenoid proteins fit these requirements. Carotenoids are widely distributed natural pigments which play important roles in photosynthesis, nutrition and illness prevention. They have a protective role in photosynthetic and non-photosynthetic organisms including humans by serving as protective colorants or by quenching singlet oxygen (^1^O_2_) and free radicals induced by exogenous sensitizers or produced by metabolic processes (reviews^1^,^2^,^3^,^4^). Carotenoids which are relatively hydrophobic molecules, typically occur solubilized in membranes or non-covalently attached to membrane or soluble proteins. In photosynthetic organisms, they are mainly found in the membrane-embedded, chlorophyll-containing-antennae where they have the dual activities of harvesting solar energy and quenching excess energy and ^1^O_2_ (see reviews^1^,^5^). A number of water soluble carotenoid proteins from photosynthetic organisms have also been characterized^6^,^7^,^8^. The cyanobacterial Orange Carotenoid Protein (OCP) is one of the best characterized soluble carotenoid proteins. We have recently demonstrated that the OCP is an excellent antioxidant--better than vitamin C, trolox, tocopherol and isolated carotenoids^9^. The OCP protects cyanobacteria by quenching the ^1^O_2_ formed in reaction centers and antennae^9^. It was first described by Holt and Krogmann in 1981^10^ and is present in the majority of cyanobacteria containing phycobilisomes (PBs), the large extra-membrane antenna formed by blue and red phycobiliproteins^11^.

The OCP is a photoactive protein^12^; it is essential for a photoprotective mechanism that decreases the excitation energy arriving at photochemical reaction centers^13^. The OCP has an α-helical N-terminal domain (residues 15–165) and an α/β C-terminal domain (residues 190–317) (Fig. 1A)^14^. The carotenoid, 3′-hydroxyechinenone (hECN), spans both domains which are joined by a flexible linker. The presence of a ketocarotenoid is essential for OCP photoactivity^15^. Light absorption by the carotenoid induces conformational changes in the carotenoid and in the protein that are essential for its photoprotective function^12^ (Fig. 1B). In darkness, the OCP is orange (OCP^o^); upon illumination, it becomes red. The red form (OCP^r^) is the active form of the protein^12^,^15^. Only OCP^r^ is able to bind the PBs. Once the OCP^r^ is bound, the carotenoid interacts with a chromophore of the PB core and quenches the excitation energy^16^,^17^,^18^. This photoprotective mechanism is activated by blue-green light but not by orange or red light that are not absorbed by the carotenoid. However, the OCP photoprotects cyanobacteria from strong orange-red light; this protection is related to the ^1^O_2_ quenching activity of the OCP^9^.

Due to the outstanding antioxidant properties of carotenoids and their role in human health, substantial effort has been devoted to the engineering of noncarotenogenic bacteria to produce high quantities of these colorants; this also requires the development of methods to isolate the carotenoids from the engineered microorganisms (reviews^19^,^20^,^21^,^22^). For many applications, a water soluble antioxidant is needed. The soluble OCP which is an excellent ^1^O_2 _quencher, is an ideal candidate. However, to-date there are no reports showing that it is possible to insert a carotenoid molecule in a protein in *E.coli*. To-date, genes encoding carotenoid proteins have been expressed in *E.coli* to isolate the apo-protein (protein without carotenoid) and then the carotenoid is attached to the apo-protein by *in vitro* reconstitution (examples^20^,^23^,^24^,^25^). The OCP has been isolated from the WT cyanobacterial strains *Synechocystis* PCC 6803 (thereafter *Synechocystis*) and *Arthrospira maxima* or from *Synechocystis* mutants overexpressing WT or mutated OCPs^12^,^14^,^26^,^27^,^28^. This, however, is a labor-intensive process because of the low concentration of the OCP in cyanobacterial cells. Indeed, even when using OCP-overexpressing strains purification requires 3 weeks to obtain 40 mg protein from 30 L of cyanobacteria cells (A Wilson and D Kirilovsky, unpublished data).

Here we describe the construction of *E.coli* strains that are able to synthesize large amounts of OCP homologs from different cyanobacterial strains incorporating various carotenoids *in vivo*. This fast holo-OCP production has already enabled us to further understand the function of different carotenoids in OCPs, for example that canthaxanthin-OCPs are very good energy and ^1^O_2_ quenchers. The work described here is important not only to accelerate the elucidation of the OCP photoprotective mechanism by rapid synthesis of variant OCPs, but promises to enable the isolation and characterization of other carotenoid proteins with potential applications for promoting human health.

## Results

### Biosynthesis of His-tagged holo-OCPs in *E.coli* cells

The aim of our work was to synthesize holo-OCPs (OCPs attaching one carotenoid molecule) from *Synechocystis*, *Arthrospira* and *Anabaena* strains in *E.coli* cells. For this purpose, the genes coding for enzymes involved in the synthesis of the desired carotenoids (supplementary Fig. 1) and the *ocp* gene must be expressed in the same cell. It is known that in *Synechocystis* and *Arthrospira* OCP binds hECN^10^,^14^. Previous work showed that it is difficult to obtain large quantities of this carotenoid in *E.coli* cells^29^,^30^. We decided to express the *ocp* gene in the presence of two other ketocarotenoids: echinenone (ECN) and canthaxanthin (CAN). *Synechocystis* OCP is able to bind ECN and the ECN-OCP is photoactive and induces PB fluorescence quenching^27^. In contrast, *Arthrospira* OCP weakly binds ECN^31^. Prior to this study, nothing was known about the *Anabaena* OCP. Although we did not know if CAN-OCP would be active, we hypothesized that the carbonyl groups present in CAN rings could allow photoactivity and stabilization of the carotenoid binding.

The *E. coli* cells producing holo-OCPs carried three plasmids. The first plasmid, pAC-BETA (or pACCAR16ΔcrtX)^32^,^33^, contained the *Erwinia herbicola* (or *Erwinia uredovora*) operon carrying the four genes (*crtB, crtE, crtI, crtY*), which are necessary to synthesize β-carotene. In the second plasmid the *crtO* gene of *Synechocystis* or the *crtW* gene from *Anabaena* PCC 7210 was introduced. While CrtO is a monoketolase synthesizing mostly echinenone from β-carotene^34^, CrtW is a diketolase that catalyses the formation of canthaxanthin^35^ (supplementary Fig. 1). The *ocp* genes were cloned in a third plasmid (pCDFDuet-1). In order to maintain the three plasmids within the same *E.coli* cell, the use of three different and compatible replication origins and three different antibiotic resistances was required (Fig. 2).

The operon containing the *crtB*, *crtE*, *crtI* and *crtY* genes was under the control of the constitutive *crtE* promoter. Thus, the β-carotene was constitutively synthesized in the *E.coli* cells. The *crtO* and *crtW* genes were under the control of the arabinose inducible promoter araBAD. The transcription of *ocp* genes was controlled by the IPTG-inducible T7lac promoter. A sequential induction of these genes was essential to isolate high quantities of holo-OCP. The expression of *crtO* or *crtW* genes was induced in *E.col*i containing a relatively high concentration of β-carotene. Subsequently the expression of *ocp* genes was induced in *E.coli* cells containing high concentrations of ECN or CAN in their membranes.

The *E. coli* cells carrying the *crtO* gene contained 15–25% of β-carotene, 70–80% ECN and 4–6% CAN. When the *ocp* genes were expressed in the presence of ECN, a mixture of apo- and holo-OCPs was obtained in all cases, but the proportion of holo-OCP varied (Table 1). The presence of photoactive holo-OCPs was already detected *in vivo*. Orange cells containing holo-OCPs became red when they were illuminated with high intensities of white light (Fig. 3A). In contrast, yellow and orange *E.coli* cultures containing only genes involved in β-carotene and ECN (without the *ocp* gene) did not change colour upon illumination (Fig. 3A). Once the cells were broken, a notable difference in the colour of the supernatants (soluble fraction) was observed. The supernatant derived from *E. coli* cells synthesizing only carotenoids, because the *ocp* gene was not induced, was clear (Fig. 3Bb) and the orange colour was concentrated in the membranes (Fig. 3Ba). The slight colour observed in the supernatant is attributable to a leak of the T7 promoter and the presence of a small concentration of OCP. In contrast, in the cells in which the *ocp* gene was induced, the supernatant was distinctly orange (Fig. 3Bc) indicating the presence of high concentrations of holo-OCPs. Moreover, under illumination the supernatant became red (Fig. 3Bd). Nevertheless, the membranes remained orange indicating that the presence of apo-proteins was not related to a lack of ECN in the cells.

The first *Synechocystis* *ocp* overexpressed genes contained an addition of 18 nucleotides coding for six His just before the stop codon (Syn-Ctag) or an extension of 45 nucleotides after the first ATG (Syn-pDuet) (supplementary Fig. 2 and Table 1). This extension which includes the sequence coding for six-His is already included in the commercial pCDFDuet-1 plasmid. Analysis of the resulting two isolated *Synechocystis* OCPs revealed that the addition of the N-terminal extension of 45 nucleotides increased the total amount of OCP present in the cells (from 4–6 mg/L to 18–22 mg/L) and the yield of holoOCP (from 25–40% (Syn-Ctag-ECN) to 35–45% (Syn-pDuet-ECN)) (Table 1).

**Figure 1:**
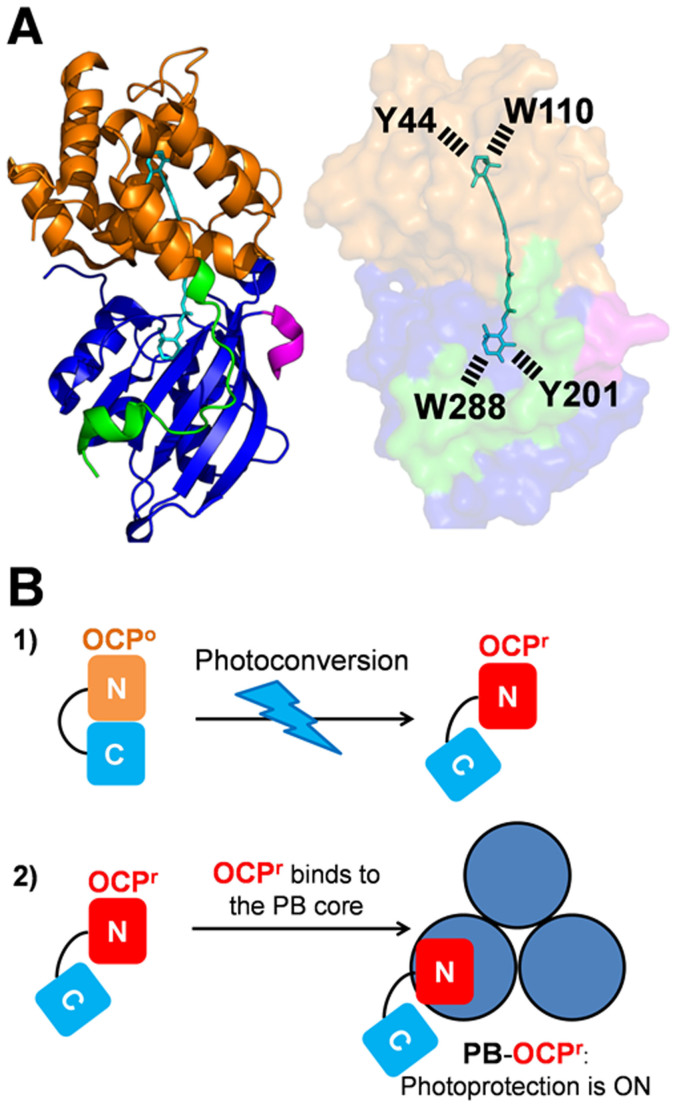
The OCP and photoprotection. (A) Structure of the OCP from *Synechocystis* PCC 6803 (Protein Data Bank ID: 3MG1) The OCP monomer in the OCP^o^ state. The N-terminal arm (residues 1–22) (green) interacts with the C-terminal domain (residues 196–315) (blue). The C-terminus is colored in rose. The N-terminal domain (residues 22–165) is orange. The hydroxy-echinenone (hECN) spans both domains of the protein. Tyr201 and Trp288 of the C-terminal domain hydrogen bond to the carbonyl group of hECN. Tyr44 and Trp110 of the N-terminal domain interact with the hydroxyl ring of hECN. The OCP was modified adding amino acids in the N-terminus (green) and/or the C-terminus (rose). (B) Model of the OCP-related photoprotective mechanism. 1) Upon light absorption the orange OCP^o^ is converted into the active open red OCP^r^. 2) OCP^r^ binds to the PB core and induces fluorescence and excitation energy quenching.

**Figure 2:**
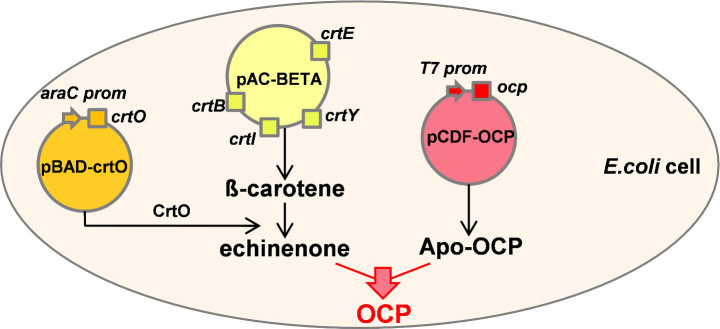
Schematic representation of OCP production in *E.coli*. *E.coli* producing ECN-OCP contain three plasmids: pAC-Beta (yellow) carries the genes involved in β-carotene synthesis (*crtB, crtE, crtI and crtY*) under the control of the *crtE* promoter; pBAD-crtO (orange), carries the *crto* gene under the control of ara promoter (arabinose inducible); CrtO enzyme converts β-carotene in echinenone; pCDF-OCP carries the *ocp* gene under the control of an IPTG inducible promoter (red).

**Figure 3:**
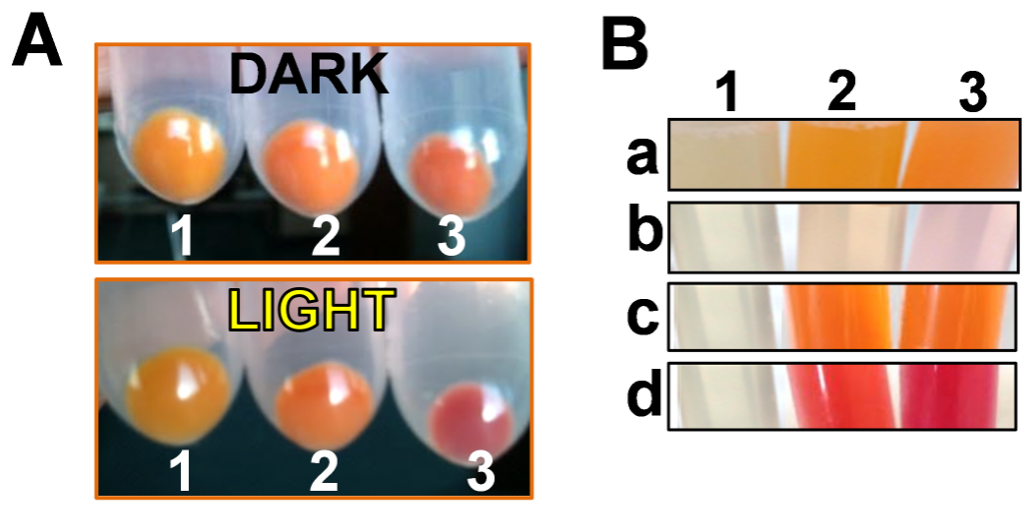
Photoactivity of the OCPs produced in *E.coli*. (A) Photoactivity observed in cells. Cell pellets carrying the three plasmids required for the synthesis of OCP in darkness or after 5 min illumination (5000 μmoles photons m^−2^ s^−1^ of white light): cells pellet without any induction (1), cells after arabinose induction producing ECN (2), cells after induction with both, arabinose and IPTG, producing holo-OCP (3). (B) Location of carotenoids and OCPs in *E.coli* cells: (a) membrane and (b) soluble fractions of *E.coli* cells without plasmids (1) or with the three plasmids and after arabinose induction, synthesizing ECN (2) or CAN (3). (c) and (d) show the soluble fractions of cells producing ECN-OCP (2) and CAN-OCP (3) in darkness (c) and after illumination (d).

Since a modification on the N-terminus seemed to increase the yield of holoOCPs in *E.coli* cells, other *ocp* modifications were tested. In all cases (with the exception of one), nine to 45 nucleotides coding for a series of non-charged or charged amino acids were added just after the first ATG in the *ocp* gene containing a sequence coding for six-His in its 3′ end (supplementary Fig. 2 and Table 1). In the construction lacking the C-terminal His-tag, 27 nucleotides including the sequence coding for six-His were added to the 5′ end (Syn-3aaNtag) (supplementary Fig. 2 and Table 1). Analysis of the isolated *Synechocystis* OCPs showed that addition of 8 to 10 amino acids largely increased the yield of holo-OCP. More than 95% of isolated *Synechocystis* OCP contained a carotenoid molecule. When only 3 to 6 amino acids were added, the yield of holoprotein also increased but slightly less (Table 1). Finally, addition of 9 amino acids, including 6 His, in the N-terminus, in the absence of C-terminal His-tag (Syn-3aaNtag) allowed the isolation of the largest quantity of holo-OCP containing almost no apo-protein: 30–35 mg holo-OCP (Table 1). All of the isolated *Synechocystis* holo-OCPs contained more than 95% ECN with only traces of CAN (supplementary Fig. 3). These results suggested that a slight destabilisation of the OCP N-terminal arm is necessary to increase and/or to stabilize OCP carotenoid binding. In the OCP^o^, this arm interacts with the C-terminal domain and seems to stabilize the closed structure of the orange form^14^,^27^ (Fig. 1).

*Arthrospira* and *Anabaena* *ocp* genes, containing sequences coding for an His-tag in the N- or the C-terminus, were also expressed in *E.coli* cells synthesizing ECN. Although the His-tag in the N-terminus increased the yield of holo *Anabaena* and *Arthrospira* OCPs (to 60 and 40%, respectively), still a large amount of apo-OCP was present (Table 1). Since the membranes remained coloured indicating the presence of ECN, the low concentration of holo-OCPs was not related to insufficient carotenoid production. The holo-*Anabaena* and holo-*Arthrospira* OCPs contained mostly ECN with traces of CAN (supplementary Fig. 3).

In an attempt to increase the yield of *Arthrospira* and *Anabaena* holo-OCPs, the *ocp* genes were induced in *E.coli* cultures synthesizing CAN. This strain, carrying the *crtW* gene, contained 15–25% of β-carotene, 50–60% CAN, 7–9% ECN and 5–7% of an unknown carotenoid. Indeed, the presence of CAN increased the yield of holoprotein to 60% in the case of *Arthrospira* OCP. In contrast, the presence of CAN decreased the yield of holo-*Synechocystis*-OCP (Syn-3aaNtag-CAN) to 75–85% and of holo *Anabaena*-OCP (Ana-3aaNtag-CAN) to 40–45% (Table 1). While the holo-*Anabaena*-CAN-OCP contained mostly CAN with only traces of ECN, holo-*Synechocystis*-CAN-OCP contained 50–70% CAN and holo-*Arthrospira*-CAN-OCP contained only 50–55% CAN (supplementary Fig. 3). Our results indicated that the binding and/or the stability of carotenoids in the protein differs between *Synechocystis* and *Arthrospira* or *Anabaena* OCPs. Most probably only the presence of hECN will allow the isolation of more than 95% of holo *Arthrospira* or *Anabaena* OCPs.

### Characteristics of the isolated OCPs

**Figure 4:**
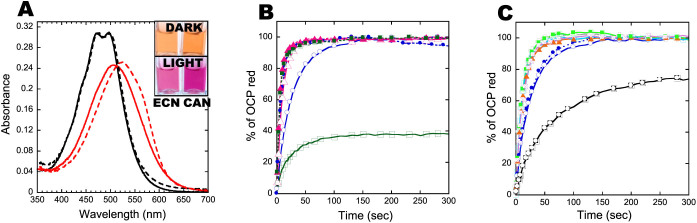
Absorbance spectra and kinetics of photoactivity of recombinant OCPs. (A) Absorbance spectra of *Synechocystis*-3aaNtag-ECN-OCP (solid line) and of *Synechocystis*-3aaNtag-CAN-OCP (dashed line). In darkness (black) or after 5 min illumination (at 18°C) with 5000 μmoles photons m^−2^ s^−1^ of white light (red). Inset: The OCPs in darkness (orange) and after 5 min illumination (red). (B) Kinetics of photoactivity (OCP^o^ to OCP^r^) at 18°C of *Syn*-3aaNtag-ECN-OCP (open circle), *Syn*-3aaNtag-CAN-OCP (closed circle), *Arthro*-3aaNtag-ECN-OCP (open triangle), *Arthro*-3aaNtag-CAN-OCP (closed triangle), *Ana*-3aaNtag-ECN-OCP (open square) and *Ana*-3aaNtag-CAN-OCP (closed square). (C) Influence of N-terminal modifications in accumulation of OCP^r^. Light-induced OCP^o^ to OCP^r^ conversion at 18°C in Tris-HCl pH8.0 (40 mM) of *Syn*-Ctag-ECN-OCP (closed circle), *Syn*-3aaNtag-ECN-OCP (open circle), *Syn*-NC15aaCtag-ECN-OCP (closed triangle), *Syn*-NC10aaCtag-ECN-OCP (open cross), *Syn*-NC8aaCtag-ECN-OCP (closed square), *Syn*-NC6aaCtag-ECN-OCP (open square), *Syn*-NC3aaCtag-ECN-OCP (square with cross), *Syn*-C8aaCtag-ECN-OCP (square with open circle). *Syn* = *Synechocystis*; *Ana* = *Anabaena*; *Arthro* = *Arthrospira*.

The isolated proteins are orange in darkness and red in strong light (Fig. 4A). Orange ECN-OCPs (OCP^o^) absorbance spectra show maxima at 472 and 496 nm and a shoulder at 450 nm, comparable to the native cyanobacteria OCPs (Fig. 4A and supplementary Fig. 4A). The absorbance spectra of CAN-OCP^o^s were slightly red shifted compared to ECN-OCP^o^s with maxima at 475 and 500 nm (Fig. 4A and supplementary Fig. 4B). The maximum of OCP^r^ absorbance spectra was at 510 nm for ECN-OCPs and 525 nm for CAN-OCPs.

All three CAN-OCPs and *Synechocystis* and *Arthrospira* ECN-OCPs completely converted to their red form (OCP^r^) under illumination (Fig. 4B). The kinetics of OCP^o^ to OCP^r^ photoconversion of the three CAN-OCPs and ECN-*Arthropira*-OCP were similar (t_1/2_ = 4–7 sec) and faster than that of the ECN-*Synechocystis* OCP (t_1/2_ = 18 sec) (Fig. 4B). The slower photoconversion of *Synechocystis* OCP compared to *Arthrospira* OCP was previously observed when the proteins were overexpressed in *Synechocystis* cells^31^. *Anabaena* ECN-OCP^o^ only partially converted to OCP^r^, suggesting slight differences in the carotenoid-protein interaction in this protein (Fig. 4B). N- and C-terminal His-tagged *Synechocystis* OCPs presented similar conversion kinetics from OCP^o^ to OCP^r^ (Fig. 4C). When both termini of the protein were modified, an acceleration of OCP^r^ accumulation was observed, suggesting a destabilization of the closed OCP^o^ (Fig. 4C). Only one exception was observed: the addition of 8 charged amino acids hindered the conversion OCP^o^ to OCP^r^ (Fig. 4C).

**Figure 5:**
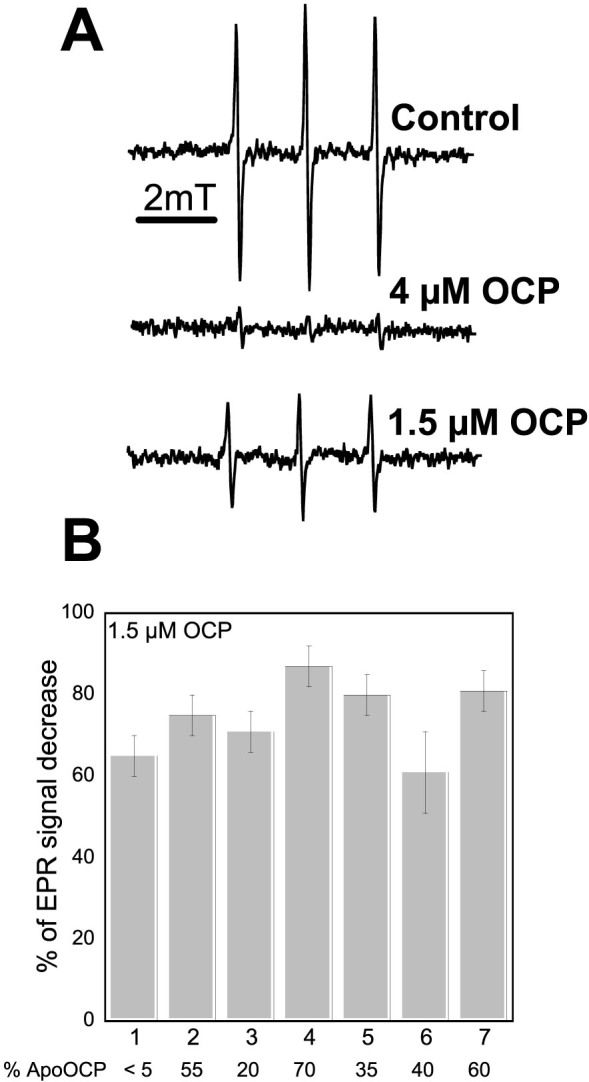
^1^O_2_ Quenching activity of the recombinant OCPs. ^1^O_2_ was produced by illumination of 10 μM methylene blue for 3 min in the presence of TEMPD-HCl and in the absence (control) or the presence of different OCPs. (A) EPR signal observed in the absence of OCP (control) or in presence of 4 μM or 1.5 μM of *Synechocystis*-3aaNtag-ECN-OCP. (B) Comparison of ^1^O_2_ quenching activity of different OCPs. The pourcentage (%) of EPR signal decrease induced by *Syn*-3aaNtag-ECN-OCP (1), *Syn*-Ctag-ECN-OCP (2), *Syn*-3aaNtag-CAN-OCP (3), *Arthro*-3aaNtag-ECN-OCP (4), *Arthro*-3aaNtag-CAN-OCP (5), *Ana*-3aaNtag-ECN-OCP (6) and *Ana*-3aaNtag-CAN-OCP (7). *Syn* = *Synechocystis*; *Ana* = *Anabaena*; *Arthro* = *Arthrospira.* The % of apo-OCP is indicated at the bottom of the figure.

The capacity of N-terminal His-tagged ECN- and CAN-OCPs to quench ^1^O_2_ was studied. Electron paramagnetic resonance (EPR) spin trapping was applied for ^1^O_2_ detection using TEMPD-HCl (2,2,6,6-tetramethyl-4-piperidone). When this nitrone reacts with ^1^O_2_, it is converted into the stable nitroxide radical, which is paramagnetic and detectable by EPR spectroscopy. The production of ^1^O_2_ was induced by illumination of the photosensitizer methylene blue. Figure 5A shows the typical EPR signal of the nitroxide radical obtained after 3 min illumination (1000 μmol photons m^−2^ s^−1^) of a solution containing methylene blue and TEMPD-HCl in the absence or presence of the OCP. The presence of only 1.5 μM holo-OCP decreased the EPR signal between 65 and 85% and 4 μM OCP quenched nearly the entire EPR signal. These results indicated that all of the *E. coli*-derived OCPs are very good ^1^O_2_ quenchers (Fig. 5B). The slight differences in the efficiency of ^1^O_2_ quenching are likely due to the presence of higher concentrations of apo-protein for the same concentration of holo-OCP (see supplementary Fig. 5). Nevertheless, our results suggested that *Arthrospira* OCP has a slightly better activity as ^1^O_2_ quencher than *Synechocystis* OCP. In contrast, *Anabaena*-ECN-OCP had a slightly lower activity as ^1^O_2_ quencher. The nature of the bound carotenoid seemed not to influence the activity, as previously suggested^9^.

**Figure 6:**
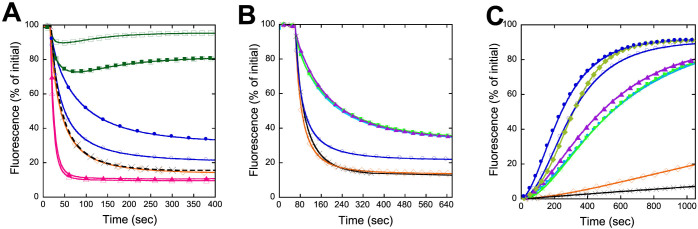
Induction of PB fluorescence quenching by OCP^r^ and fluorescence recovery WT *Synechocystis* PBs (0.012 μM) were incubated in 0.5 M phosphate buffer with pre-converted OCP^r^ (0.48 μM) during 5 min under blue-green light illumination (900 μmoles photons m^−2^ s^−1^). (A and B) Fluorescence quenching under illumination and (C) fluorescence recovery in darkness. In (A) Native *Synechocystis* OCP (cross), *Syn*-Ctag-ECN-OCP (open diamond), *Syn*-3aaNtag-ECN-OCP (open circle), *Syn*-3aaNtag-CAN-OCP (closed circle), *Ana-*3aaNtag-ECN-OCP (open square), *Ana-*3aaNtag-CAN-OCP (closed square), *Arthro-*3aaNtag-ECN-OCP (open triangle), *Arthro-*3aaNtag-CAN-OCP (closed triangle). In (B) Native *Synechocystis* OCP (cross), *Syn*-3aaNtag-ECN-OCP (open circle), *Syn*-Ctag-ECN-OCP (open diamond), *Syn*-NC6aaCtag-ECN-OCP (closed circle), *Syn*-NC8aaCtag-ECN-OCP (closed square), *Syn*-NC10aaCtag-ECN-OCP (closed triangle). In (C) Native *Synechocystis* OCP (cross), *Syn*-Ctag-ECN-OCP (open diamond), *Syn*-NC8aaCtag-ECN-OCP (closed square), *Syn*-NC10aaCtag-ECN-OCP (closed triangle), *Syn*-3aaNtag-ECN-OCP (open circle), *Syn*-3aaNtag-CAN-OCP (closed circle), *Syn*-pDuet-ECN-OCP (closed diamond).

Finally, the ability of the OCPs to quench PB fluorescence was tested. *Arthrospira* and *Synechocystis* OCPs isolated from *E coli* were able to induce a large PB fluorescence quenching (Fig. 6A and supplementary Fig. 6A). *Arthrospira* OCPs induced the fastest kinetics of fluorescence quenching and *Anabaena* OCPs the slowest, as previously observed with native OCPs (Ref. 31 and supplementary Fig. 6C). The activity of *Synechocystis* ECN-OCP was higher than that of *Synechocystis* CAN-OCP; the opposite was observed with *Anabaena* OCP since ECN-OCP was only partially converted to the red form (Fig. 6A). Addition of 6 to 10 amino acids to the N-terminus of the C-terminal His-tagged OCP partially inhibited the fluorescence quenching, suggesting that OCP binding to PBs is hindered (Fig. 6B and supplementary Fig. 6B). The weaker binding of these modified OCPs was confirmed by the rapid fluorescence recovery observed when PBs-OCP complexes were incubated in darkness (Fig. 6C). In contrast, C-terminal His-tagged OCP remained mostly attached to the PBs (Fig. 6C). Both N- and C-terminal His-tagged *Synechocystis* ECN-OCPs isolated from *E.coli* were able to induce a large fluorescence quenching (Fig. 6A). However, a fast fluorescence recovery was observed only with *Synechocystis* N-terminal His-tagged OCPs (Fig. 6C). Thus, the behaviour of OCPs is affected by the location of the His-tag.

## Discussion

The use of soluble carotenoproteins as antioxidants to promote human health is an area of active research and, consequently, methods to produce them in high yields are important. The aim of our work was to synthesize holo-OCPs (OCPs attaching the carotenoid) in *E.coli* to develop a method for obtaining high quantities of carotenoproteins. Using the method described here, we obtained 200 times more holo-OCP in 20% of the time of previously established purifications involving overexpression in cyanobacterial cells (C Bourcier de Carbon, A Wilson and D Kirilovsky, unpublished data). In only four days more than 30 mg holo-*Synechocystis* OCP can be obtained from 1 L of *E.coli* cells using the construction Syn-3aaNtag-ECN.

The key elements of this production method are the choice of promoters and the sequential induction of genes. β-carotene must be present in the membrane before induction of *ctrO* or *crtW* genes leading to the synthesis of ECN or CAN, respectively. More importantly, ECN and CAN have to be synthesized in advance and present in the membrane before induction of the *ocp* gene. The presence of IPTG in the growth medium inhibits cell growth even at low concentrations. In addition, the T7lac promoter cannot be induced at temperatures higher than 30°C. In contrast, arabinose enhances cell growth and the araBAD promoter allows induction at 37°C. Thus, the carotenoid genes must be induced first with arabinose at 37°C to obtain a high concentration of carotenoid- containing cells and then, the carotenoprotein gene could be induced by IPTG addition at lower temperatures (20 to 28°C) to slow down protein synthesis, allowing protein folding and carotenoid binding.

The possibility of isolate holo-OCPs from *E.coli* cells constitutes a major advance for the investigation of the molecular mechanism of OCP since it facilitates rapid isolation of mutant proteins with new characteristics. The method has already permitted us to further characterize OCPs revealing different phenotypes related to specificity and strength of carotenoid binding. We demonstrate here that all OCPs are able to bind CAN and that CAN-OCPs are photoactive and able to induce large PB fluorescence quenching, like the native hECN-OCPs. This demonstration was not previously possible when OCPs were isolated by overexpression in *Synechocystis* cells since they contain only traces of CAN.

We also show here that *Arthrospira*, *Anabaena* and *Synechocystis* OCPs are characterized by different phenotypes in terms of specificity and strength of carotenoid binding. *Synechocystis* OCP binds and stabilizes ECN better than CAN. In contrast, *Arthrospira* and *Anabaena* OCPs preferentially bind CAN over ECN and have a low affinity for both carotenoids. It is difficult to explain these differences based in the comparison of *Arthrospira* and *Synechocystis* OCP^o^ structures due to the high sequence identity among amino acids forming the carotenoid-binding pocket and the similar carotenoid orientation in the proteins (supplementary Fig 9A). Nothing is known about how the carotenoid is introduced in the apo-OCP. We can hypothesize that the OCP is synthesized by membrane bound ribosomes and that the carotenoid is introduced during the synthesis of the N-terminal domain. It could be possible that the amino acids involved in this initial binding differ from those in the carotenoid-binding pocket of mature OCP^o^. Many of the amino acid substitutions among the three OCPs are localized on the outer surface of the N-terminal domain and could have a role in primary binding (supplementary Fig 9B and 9C).

The most unexpected result was the different behavior in PBs binding of *Synechocystis* OCPs with a His-Tag on the N-terminus compared to those with the tag on the C-terminus. In the past, all OCP characterizations and the construction of stable OCP-PBs complexes were made with isolated C-terminal His-tagged OCPs^12^,^16^,^28^. These OCPs, once bound to PBs at 0.5 or 0.8 M phosphate, remain almost permanently attached and the PBs remain quenched^16^,^28^. This characteristic allowed the isolation of quenched OCP-PB complexes^16^,^28^. Here, we show that the absence of the C-terminal His-tag largely accelerates the dark recovery of PB fluorescence, suggesting a decreased stability of bound OCP^r^. In contrast, OCP^r^ binding kinetics were only slightly affected. In addition, a longer N-terminus in C-terminal His-tagged OCPs hinders the OCP binding and destabilizes the strong OCP^r^ attachment to PBs. Our results strongly suggest that while the C-terminal His-tag increases the stability of OCP^r^-PB complexes, a longer N-terminus destabilizes this attachment.

Production of antioxidant molecules and proteins is a topic of considerable general interest to plant and human biologists since oxidative stress is involved in many processes leading to cell death or tissue damage. Here we show that all three recombinant OCPs show excellent activity as ^1^O_2_ quencher. Other soluble carotenoproteins also display good antioxidant properties like Asta P^6^ and could be used in nutraceutics, cosmetics, etc. The possibility of engineering noncarotenogenic bacteria to produce carotenoproteins, like the OCP, which are present at relative low concentrations in the native organisms, constitutes a major breakthrough in efforts to obtain large quantities of carotenoid molecules as antioxidants.

In addition, the ability to synthesize holo-OCPs in *E.coli* is an important step in the construction of a biofuel (biomass) producing minimal microbe using sunlight as natural source of energy. In order to create these minimal entities new synthetic reaction centers containing the minimal number of components needed for electron transport are being constructed and antenna molecules are being attached to them to expand the spectral range for light absorption (for example^36^,^37^,^38^,^39^,^40^). Presently, nothing is done to protect these systems. The OCP, a good quencher of excitation energy and singlet oxygen, is an excellent candidate for this function. It can regulate the excitation energy arriving to the reaction centers and quench the singlet oxygen formed by the inevitable secondary, dangerous reactions.

## Methods

### Amplification and cloning of *Crt* genes encoding enzymes involved in carotenoid synthesis and of *ocp* gene

The plasmids pAC-BETA (gift of Prof F. Cunningham) and pACCAR16ΔcrtX (gift of Prof G. Sandmann), which contain a P15A origin of replication and the *crtB*, *crtE*, *crtI* and *crtY* genes under the control of the promoter of *crtE* from *Erwina herbicola* and *Erwina uredovora* respectively, were used^32^,^33^. All results presented in this article were obtained with pAC-BETA.

The *crtO* and *crtW* genes were cloned into a modified Plasmid pBAD/gIII A (from Invitrogen) which contains a PBR322 origin of replication, an arabinose inducible promoter (araBAD) and an ampicillin resistance marker. The Plasmid pBAD/gIII A was first modified to avoid the export of the recombinant protein into the periplasmic space of the cells. For this purpose, the region encoding the “gene III signal sequence” was deleted. Primers used for the PCR mutagenesis were pBAD/gIIIAmut (F and R) (supplementary Fig. 7). The modified plasmid pBAD/gIII A was named pBAD. The Plasmid pBAD was digested with BgIII and EcoRI restriction enzymes to clone the *crtO* gene (*slr0088*) of *Synechocystis* PCC6803 or with NcoI and EcoRI restriction enzymes to clone the *crtW* gene (*alr3189*) of *Anabaena* PCC7120. Primers CrtO (F and R) and CrtW (F and R) were used to amplify *crtO* and *crtW* genes respectively (supplementary Fig. 7). The resulting plasmids were named pBAD-CrtO and pBAD-CrtW.

The *ocp* gene was cloned in the plasmid pCDFDuet-1 (from Novagen). The plasmid pCDFDuet-1 contains a CDF origin of replication, T7lac promoter and Streptomycin/Spectinomycin resistance. The sequences of synthetic oligonucleotides (primers) used in the amplification and modification of all the genes are described in supplementary Fig. 7.

### N-terminal His-tagged OCP

The pCDFduet-1 plasmid was digested with EcoRI and NotI to clone the different *ocp* genes (*slr1963* from *Synechosystis* PCC6803, *NIES39_N00720* from *Arthrospira Platensis* PCC7345 and *all3149* from *Anabaena* PCC 7120). The primers OCPSyn-pDuet (F and R) were used to amplify the *Synechocystis ocp* gene (1104 nucleotides) using genomic DNA of *Synechocystis* PCC6803 as template. The primers OCPAna-pDuet (F and R) were used to amplify the *Anabaena ocp* gene (1076 nucleotides) using genomic DNA of *Anabaena* PCC 7120 as template. The primers OCPArthro-pDuet (F and R) were used to amplify the *Arthrospira ocp* gene (1355 nucleotides) using the plasmid pOF7345 as template^31^. The resulting PCR products were introduced into pCDFDuet-1 to create the pCDF-OCPSyn-pDuet, pCDF-OCPArthro-pDuet and pCDF-OCPAna-pDuet plasmids. In the OCP isolated from *E.coli* strains carrying these plasmids, an extension of 15 amino acids was present in the N-terminal of the OCP protein. This extension contains a His-tag comprising 6 His residues.

### C-terminal His-tagged OCP

To obtain a C-terminal His-tagged *Synechocystis* OCP, it was first, necessary to abolish a NcoI site in the *Synechocystis ocp* gene sequence; accordingly the GCC sequence coding for Ala73 was changed to GCG (also coding for an alanine) using the plasmid pSK-OCPsyn-CterHisTagΔFRP^12^. Then, pCDFDuet-1 was digested with NcoI and NotI to excise the N-terminal extension containing the His-tag initially present in this plasmid. The *ocp* genes containing a C-terminal His-Tag from *Synechosystis* PCC6803, *Arthrospira* *Platensis* PCC7345 and *Anabaena* PCC 7120 were cloned in the plasmid. The primers OCPsyn-Ctag (F and R) were used to amplify the *ocp* gene tagged in C-terminal domain from the plasmid pSK-OCPsyn- CterHisTagΔFRP- A73A^12^. The primers OCParthro-Ctag (F and R) were used to amplify the *ocp* gene from the plasmid p2A7345His^31^. The primers OCPana-Ctag (F and R) were used to amplify the *ocp* gene from genomic DNA of *Anabaena* PCC 7120, the C-terminal His-tag was then added by PCR mutagenesis. The resulting PCR products were introduced into pCDFDuet-1 to create the pCDF-OCPsyn-Ctag, pCDF-OCParthro-Ctag and pCDF-OCPana-Ctag plasmids.

### Modifications in His-tagged OCPs

The sequences added after the first ATG of the *ocp Synechocystis* gene are described in supplementary Fig. 2. The modifications (NC15, NC10, NC8, NC6, NC3, Mix15 and C8, Table 1) were introduced by directed mutagenesis, using the pCDF-OCPSyn-Ctag plasmid as template and the different oligonucleotides described in supplementary Fig. 7. The modification 3aaNtag (Table 1) was created by site-directed mutagenesis using the pCDF-OCPSyn-pDuet plasmid as template and the oligonucleotides described in supplementary Fig. 7. This mutagenesis causes the deletion of part of the OCP N-terminal extension present in the pCDF-OCPSyn-pDuet plasmid. The modification 3aaNtag was also created in the *ocp* genes of *Arthrospira* and *Anabaena* using the pCDF-OCParthro-pDuet and pCDF-OCPana-pDuet plasmids as templates and the oligonucleotides described in supplementary Fig. 7.

### Transformation of *E.coli* cells and induction of genes

*E.coli* BL21-Gold (DE3) cells from Agilent Technologies (F– ompT hsdS(rB – mB–) dcm+ Tetr gal λ(DE3) endA Hte) were used for OCP production. BL21 cells were transformed simultaneously with three plasmids: pAC-BETA, pBAD-CrtO (or pBAD-CrtW) and pCDF-OCP. The pCDF-OCP plasmid contains WT or modified sequences of *ocp* genes. The transformed *E.coli* cells were grown in the presence of three antibiotics (ampicillin (50 μg/ml), chloramphenicol (17 μg/ml) and streptomycin (25 μg/ml)) to maintain the three different plasmids in the same *E.coli* cell. For induction of the different genes, transformed *E.coli* were grown in TB medium at 37°C for 3–4 hours until OD_600_ = 0.6. Then arabinose was added (0.02%) and the culture was grown overnight at 37°C. In the morning the cells are diluted with fresh medium and Arabinose 0.02% and they are grown at 37°C till OD_600_ = 1–1.2. Then isopropyl b-D-thiogalactoside (IPTG) (0.2 mM) was added and the cells incubated overnight at 28°C. In the morning, the cultures were harvested and pellets were stored at −80°C until they were used.

### OCP isolation and calculation of holo-OCP concentration

*E.coli* frozen cells were resuspended in the lysis buffer containing 40 mM Tris pH 8, 10% glycerol and 300 mM NaCl and were broken in dim light using a French Press. The membranes were pelleted and the supernatant was loaded onto a nickel column (Ni-Probond resin, Invitrogen). The OCP was eluted with 200 mM Imidazol. For isolation of *Synechocystis*, *Arthrospira* and *Anabaena* OCPs overexpressed in *Synechocystis*, an initial 50 mL *Synechocystis* culture was daily diluted during 2 weeks until reaching 30 L culture at OD800 = 0.8. The cells were precipitated and broken using a French Press. The OCP was isolated from the supernatant using two columns as described in^12^. Complete isolation took three weeks.

Total OCP concentration was measured using the Bradford method. At least five independent Bradford measurements of each isolated OCP were done. The concentration in mg/mL obtained by this method was converted to molar concentration using a MW of 35 kDa for the OCP. Holo-OCP concentration was calculated based in the fact that each holo-OCP binds one carotenoid molecule and thus, the molar concentrations of carotenoid and holo-OCP are identical. Carotenoid concentration was first calculated in mg/mL from the carotenoid absorbance at 496 nm and using A^1%^_1 cm_ = 2158 and then converted to molar concentration. The ratio between the molar concentration of holo-OCP and total OCP gives the percentage of holo-protein described in Table 1. When this ratio is around 1, we estimated that the preparation contained 100% holo-protein.

### Absorbance spectra and photoactivity kinetics of OCP

Absorbance spectra and kinetics of photoactivity (illumination with 5000 μmol photons m^−2^ s^−1^ of white light) and dark recovery were measured in a Specord S600 (Analyticjena) at 18°C.

### Measurements of OCP fluorescence quenching activity

Isolated *Synechocystis* PBs in 0.5 or 0.8 M phosphate were incubated in the presence of different modified OCP^r^s under illumination with strong blue-green light. The high concentration of phosphate was needed to maintain the integrity of PBs. Phosphate also influences the strength of OCP binding to PBs; it is stronger at 0.8 M than at 0.5 M phosphate^16^. The ratio of OCP to PB was 40 in all the experiments as previously described^16^,^18^. The concentration of the OCP for these experiments was calculated from the carotenoid absorbance spectra since only the OCP attaching a carotenoid is able to be photoactivated and to bind to PBs. The presence of apo-OCP did not hinder OCP^r^ binding (supplementary Fig. 8).

Fluorescence quenching and recovery were monitored with a pulse amplitude modulated fluorometer (101/102/103-PAM; Walz, Effelrich, Germany). The fluorescence quenching was induced by 900 μmol photons m^−2^ s^−1^ of blue-green light (400–550 nm). All measurements were carried out at 23°C in 0.5 or 0.8 M phosphate buffer. The OCP was pre- converted to the red form by 10 min illumination with 5000 μmol photons m^−2^ s^−1^ of white light at 4°C.

### ^1^O_2_ detection by EPR spin trapping

Electron paramagnetic resonance (EPR) spin trapping was applied for ^1^O_2_ detection using TEMPD-HCl (2,2,6,6-tetramethyl-4-piperidone) (100 mM). When this nitrone reacts with ^1^O_2_, it is converted into the stable nitroxide radical, which is paramagnetic and detectable by EPR spectroscopy. The production of ^1^O_2_ was induced by illumination of the photosensitizer methylene blue (10 μM). The measurements were done in buffer 100 mM Tris-HCl pH 8 in the absence or in the presence of different concentrations of purified OCPs. The samples were illuminated for 3 min with white light (1000 μmol photons m^−2^ s^−1^). The EPR settings were as follows: hall center field = 3467.270 G, microwave frequency = 9.74 Ghz, power = 4.450 mV and number of scans = 12.

### Measurement of carotenoid content in OCPs

The carotenoid content of *E.coli* cells and the isolated OCPs was analysed by High-Performance Liquid Chromatography (HPLC) and Mass spectrometry as described in^9^.

## Author Contributions

C.B.C. and D.K. designed research; C.B.C., A.T., A.W. and F.P. performed research; C.B.C., A.T. and D.K. analyzed data; D.K. wrote the paper with inputs of C.B.C. and A.T.

## Supplementary Material

Supplementary Information Supplementary data

## Figures and Tables

**Table 1:** Percentage and amount of holo-OCP obtained from 1 L culture of *E.coli*. The Sequences of amino acids added to the N- or C-terminus are given

Name of OCP	Added Amino acid sequence	Total OCP (mg/L)	Holo-OCP (% of total OCP)	Holo-OCP (mg/L)
ECN – *Synechocystis* OCPs				
Syn-Ctag-ECN	-HHHHHH-StopCodon	4–6	25–40	1–5
Syn-pDuet-ECN	MGSS-HHHHHH-SQDP-	18–22	35–45	5–10
Syn-MIX15aaCtag-ECN	MGSSRLDNPEKTDIEP-	20–22	30–40	5–10
Syn-NC15aaCtag-ECN	MGSSNQANQVTLNPQV-	18–21	45–55	8–10
Syn-NC10aaCtag-ECN	MGSSNQANQVT-	9–11	>95	10
Syn-NC8aaCtag-ECN	MGSSNQANQ-	15–17	>95	15
Syn-NC6aaCtag-ECN	MGSSNQA-	19–21	78–85	15–20
Syn-NC3aaCtag-ECN	MGSS-	8–10	70–80	5–10
Syn-C8aaCtag-ECN	MGSSKKRRA-	20–22	78–85	15–20
Syn-3aaNtag-ECN	MGSS-HHHHHH-	30–35	≥95	30–35
ECN - *Anabaena* and *Arthrospira* OCPs				
Ana-3aaNtag-ECN	MGSS-HHHHHH-	50–60	50–60	25–35
Ana-Ctag-ECN	-HHHHHH-StopCodon	50–60	40–45	20–25
Arthro-3aaNtag-ECN	MGSS-HHHHHH-	25–30	30–40	5–15
Arthro-Ctag-ECN	-HHHHHH-StopCodon	15–20	10–25	1–5
CAN - OCPs				
Arthro-3aaNtag-CAN	MGSS-HHHHHH-	25–30	60–65	15–20
Arthro-Ctag-CAN	-HHHHHH-StopCodon	30–35	50–60	15–20
Syn-3aaNtag-CAN	MGSS-HHHHHH-	8–10	75–85	5–10
Ana-3aaNtag-CAN	MGSS-HHHHHH-	60–70	40–45	25–30
Ana-Ctag-CAN	-HHHHHH-StopCodon	60–70	50–60	30–45
