# Supplementary material for: Biosynthesis of soluble carotenoid holoproteins in Escherichia coli
Source: Sci Rep. 2015 Mar 13;5:9085. doi: 10.1038/srep09085 (PMC4358027; doi:10.1038/srep09085)
Supplement: Supplementary Information — Supplementary data [file srep09085-s1.pdf]

Supplementary Data

Biosynthesis of soluble carotenoid holoproteins in *Escherichia coli*

Céline Bourcier de Carbon[1,2,3], Adrien Thurotte[1,2], Adjélé Wilson[1,2], François Perreau[4,5] and Diana Kirilovsky[1,2]

Supplementary Figures S1 to S9

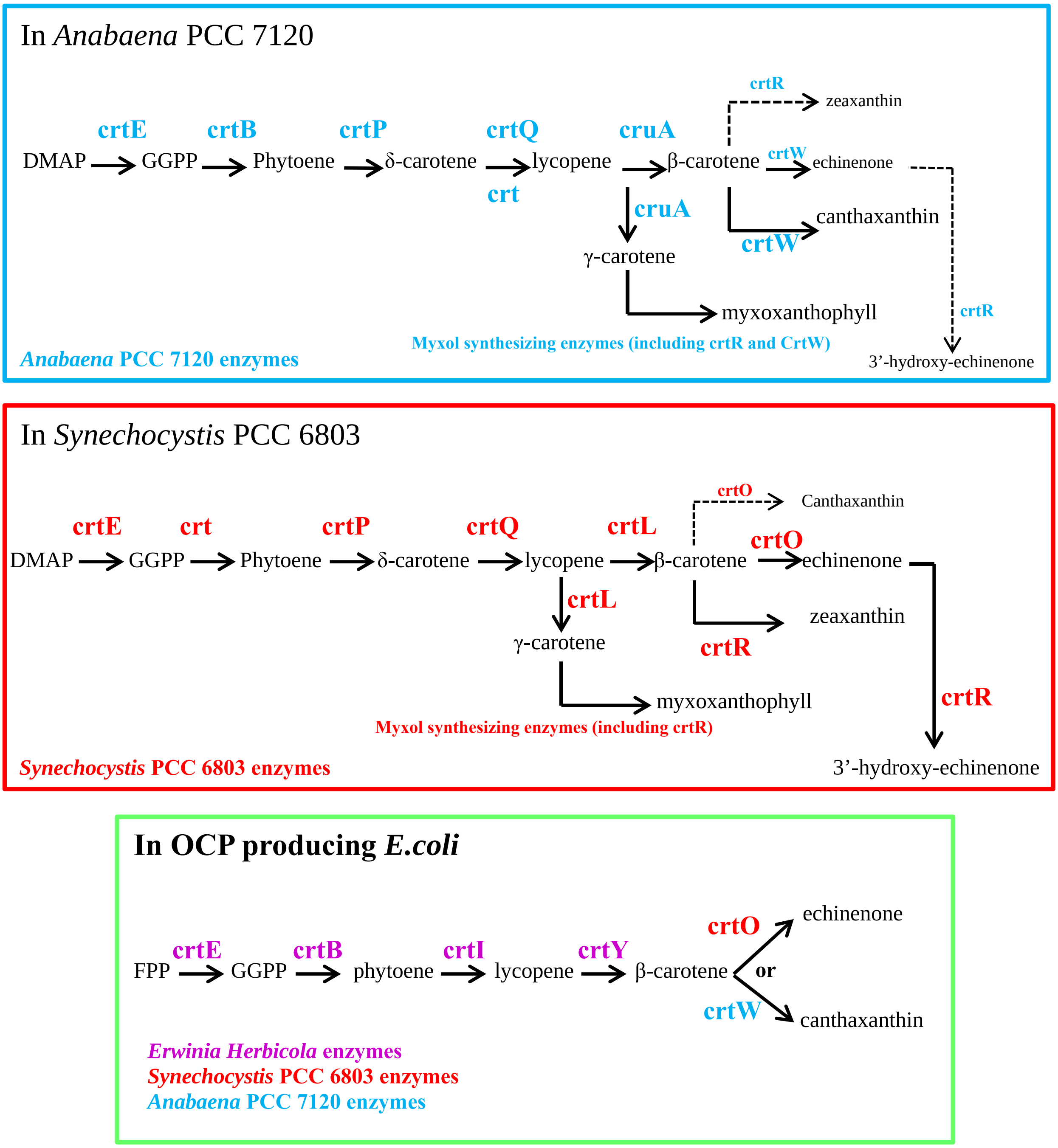

Sup Fig 1: Schematic carotenoid biosynthetic pathway. Carotenoid biosynthesis in *Synechocystis* and *Anabaena* cyanobacteria strains and in *E.coli* strains producing ECN or CAN-OCPs created in this work.

A- Sequence including the His-tag added in the C-terminus of WT OCPs
Syn-Ctag: *ocp* gene-**CACCACCACCACCACCAC**-STOPcodon

B- Sequences including the His-Tag added on the N-terminus of WT OCPs
Syn-pDuet: **ATG**GGCAGCAGC**CATCACCATCATCACCAC**AGCCAGGATCCG-*ocp* gene-STOPcodon
Syn-3aaNtag: **ATG**GGCAGCAGC**CATCACCATCATCACCAC**-*ocp* gene-STOPcodon

C- Sequences added on the N-terminus of C-terminal His-tagged *Synechocystis* OCP

Name of OCP	Sequence added on the N-terminus
Syn-NC15aaCtag (nucleotids)	**ATG**GGCAGCAGCAATCAGGCTAATCAGGTGACTCTAAATCCACAAGTT
Syn-NC15aaCtag (amino acids)	**M**GSSNQANQVTLNPQV
Syn-NC10aaCtag (nucleotids)	**ATG**GGCAGCAGCAATCAGGCTAATCAGGTGACT
Syn-NC10aaCtag (amino acids)	**M**GSSNQANQVT
Syn-NC8aaCtag (nucleotids)	**ATG**GGCAGCAGCAATCAGGCTAATCAG
Syn-NC8aaCtag (amino acids)	**M**GSSNQANQ
Syn-NC6aaCtag (nucleotids)	**ATG**GGCAGCAGCAATCAGGCT
Syn-NC6aaCtag (amino acids)	**M**GSSNQA
Syn-NC3aaCtag (nucleotids)	**ATG**GGCAGCAGC
Syn-NC3aaCtag (amino acids)	**M**GSS
Syn-MIX15aaCtag (nucleotids)	**ATG**GGCAGCAGCCGACTAGACAATCCAGAAAAAACTGACATTGAACCA
Syn-MIX15aaCtag (amino acids)	**M**GSSRLDNPEKTDIEP
Syn-C8aaCtag (nucleotids)	**ATG**GGCAGCAGCAAAAAGAGAAGGGCT
Syn-C8aaCtag (amino acids)	**M**GSSKKRRA

Sup Fig 2: **Nucleotides and amino acids sequences added to the 3' and 5' ends of *ocp* gene (N-terminus and C-terminus of *Synechocystis ocp* gene).**

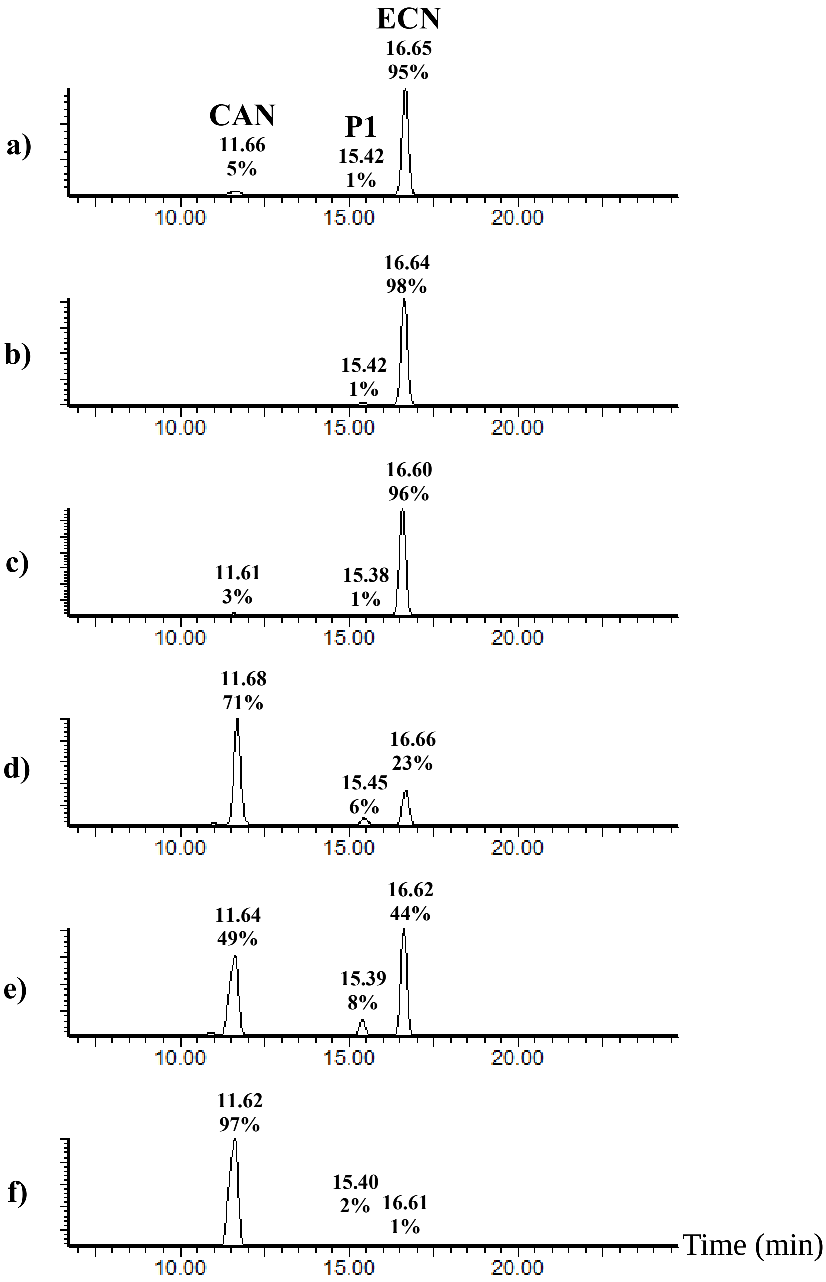

Sup Fig 3: UV Chromatograms (450 nm) of isolated OCPs from *E.coli* culture

a) *Syn*-3aaNtag-ECN-OCP, b) *Arthro*-3aaNtag-ECN-OCP, c) *Ana*-3aaNtag-ECN-OCP, d) *Syn*3aaNtag-CAN-OCP, e) *Arthro*-3aaNtag-CAN-OCP and f) *Ana*-3aaNtag-CAN-OCP. Retention time (min) and relative integration of peak area indicated (% of shown). CAN: canthaxanthin, P1: unknown carotenoid of MW 548, ECN: echinenone.

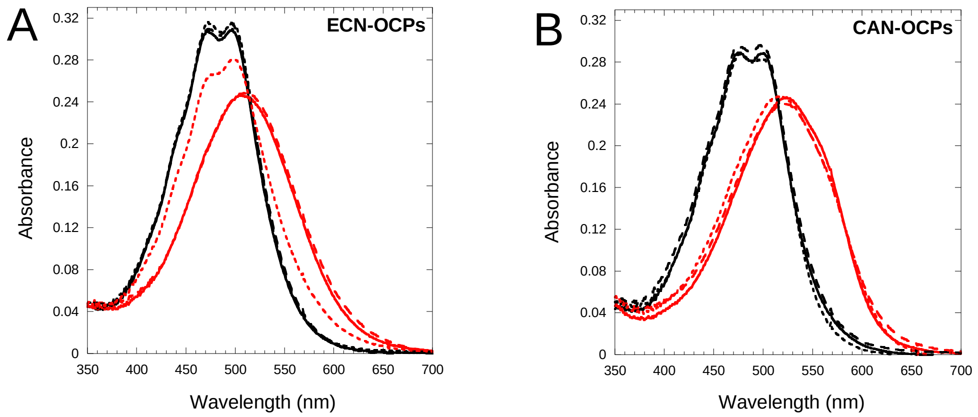

Sup Fig 4: **Photoactivity of recombinant OCPs**

(A) Absorbance spectra of the dark (black) and light (red) forms of the ECN-OCPs. OCP-*Syn*-3aaNtag-ECN (solid line), OCP-*Arthro*-3aaNtag-ECN (dashed line) and OCP-*Ana*-3aaNtag-ECN (dotted line). To obtain the spectrum of the light form, the OCP was illuminated with 5000 µmol photons m^{-2} s^{-1} of white light, at 18°C, for 5 min.

(B) Absorbance spectra of the dark (black) and light (red) forms of the CAN-OCPs. Symbols are the same as in (A).

OCP	% of EPR signal decrease	% of Apo-OCP
Syn-3aaNtag-ECN	65	< 5
Syn-3aaNtag-ECN + 20% ApoOCP	67	20
Syn-3aaNtag-ECN + 40% ApoOCP	71	40
Syn-3aaNtag-ECN + 60% ApoOCP	73	60
Syn-Ctag-ECN	72	60

Sup Fig 5: Apo-OCP influence on singlet oxygen quenching.

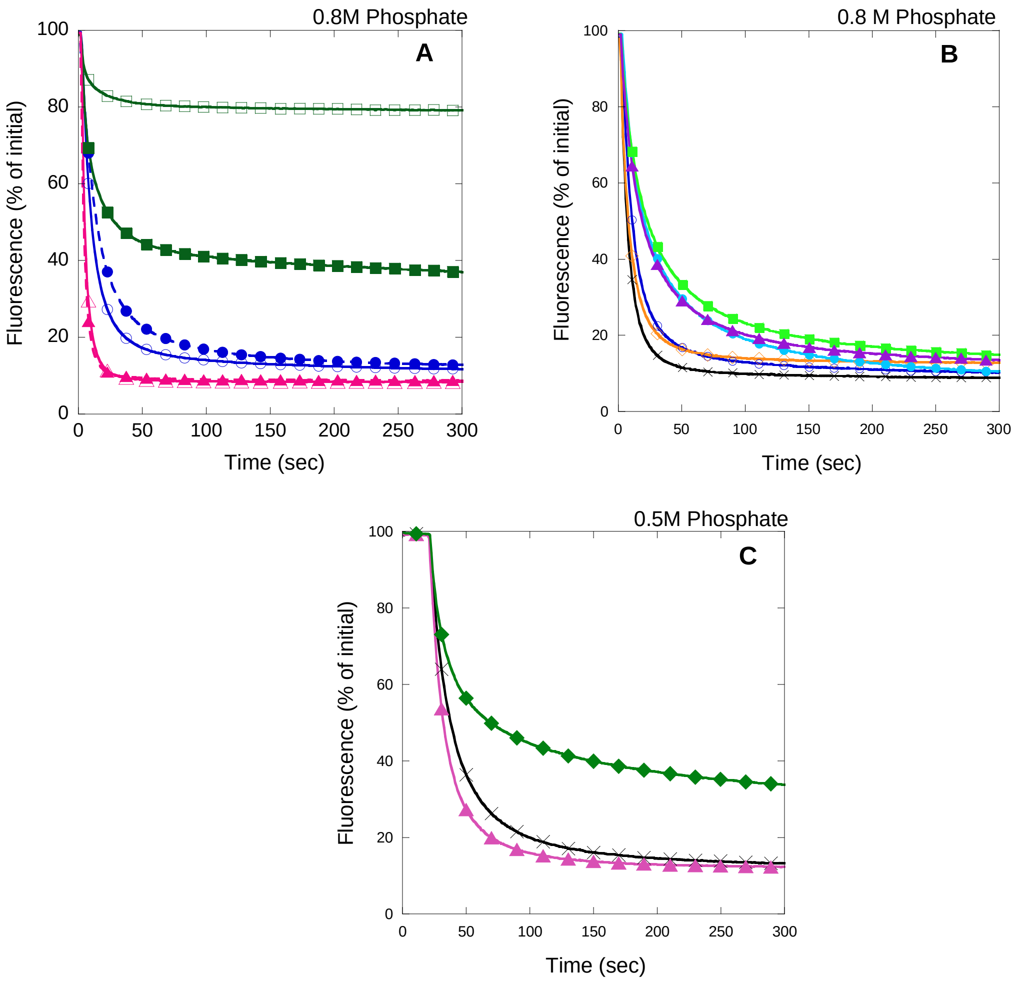

Sup Figure 6: Induction of PB fluorescence quenching by OCP^r and fluorescence recovery WT *Synechocystis* PBs (0.012 μM) were incubated in 0.8 M (A,B) or 0.5 M (C) phosphate buffer with preconverted OCP^r (0.48 μM) during 5 min under blue-green light illumination (900 µmol photons m^{-2} s$^{-1)}$.). In (A) *Syn*-3aaNtag-ECN (open circle), *Syn*-3aaNtag-CAN (closed circle), *Ana*-3aaNtag-ECN (open square), *Ana*-3aaNtag-CAN (closed square), *Arthro*-3aaNtag-ECN (open triangle), *Arthro*-3aaNtag-CAN (closed triangle). In (B) fluorescence quenching under illumination of native *Synechocystis* OCP (cross), *Syn*-3aaNtag-ECN (open circle), *Syn*-Ctag-ECN (open diamond), *Syn*-NC6aaCtag-ECN (closed circle), *Syn*-NC8aaCtag-ECN (closed square) , *Syn*-NC10aaCtag-ECN (closed triangle; in (C) Native *Ana*-OCP (closed diamond), Native *Syn*-OCP (cross) and Native *Arthro*-OCP (closed triangle). The differences in the kinetics of fluorescence quenching are bigger at 0.5 M phosphate than at 0.8M phosphate. Compare Figs 6A and 6 B with sup Figs 6A and 6B respectively.

Primer names	Primer sequences
**pBADgIII-CrtO (BglII/EcoRI)**	Cloning using pBAD/gIII A (**Invitrogen)**<br>5'-AACAGAAGATCTATCACCACCGATGTTGTC-3’<br>5'-TCTGTTGAATTCGCTTCACTTGCCATACAC-3'
**pBAD-CrtO**	Mutagenesis using pBADgIII-CrtO<br>5'-CAGGAGGAATTAACCATGATCACCACCGATGTTGTCATTATTGGGGCG-3’<br>5'-CATGGTTAATTCCTCCTGTTAGCCCAAAAAACGGGTATGGAGAAACAG-3'
**pBAD**	Mutagenesis using pBAD/gIII A<br>5'-CAGGAGGAATTAACCATGACCATGGAGCTCGAGATCTGCAGCTGGTAC-3'<br>5'-CATGGTTAATTCCTCCTGTTAGCCCAAAAAACGGGTATGGAGAAACAG-3'
**pBAD-crtW (NcoI/ EcoRI)**	**pBAD-crtW NcoI/ EcoRI**<br>Cloning using pBAD and primers :<br>5’- GATATACCATGGTTCAGTGTCAACCATCATC- 3’<br>5’- GCATCGGAATTCAGTCGTATTCCAGCAG -3’
**OCPSyn-pDuet (EcoRI/NotI)**	5'-AACAGAGAATTCTCCATTCACCATTGACTCT-3’<br>5'-TCTGTTGCGGCCGCTGTAACATTAACTTATGA-3'
**OCPSyn-Ctag**	Mutagenesis of NcoI site [GCC(A)73GCG(A)] in OCP<br>5'-CAAGGAAATTCAAGCGATGGGGCCCCTC-3’<br>5'-GAGGGGCCCCATCGCTTGAATTTCCTTG-3'<br>Amplification of the modified *ocp* gene:<br>5'-AACAGACCATGGCATTCACCATTGACTCT-3’<br>5'-TCTGTTGCGGCCGCTGTAACATTAACTTATGA-3‘
**OCPSyn-3aaNtag**	5'-CATCACCATCATCACCACCCATTCACCATTGAC-3‘<br>5'-GTGGTGATGATGGTGATGGCTGCTGCCCATGGT-3'
**OCPArthro-Ctag**	5'-AACAGACCATGGCATTCACCATTGACTCG-3‘<br>5'-TCTGTTGCGGCCGCTTATCATTACCTAATTGAGCATT-3'
**OCPArthro-3aaNtag**	5'-CATCACCATCATCACCACCCATTCACCATTGAC-3‘<br>5'-GTGGTGATGATGGTGATGGCTGCTGCCCATGGT-3'
**OCPAna-Ctag**	5'-AACAGACCATGGCAATTACTATCGATTCC-3‘<br>5'-TCTGTTGCGGCCGCGACTCAATCTTAAACTTGTA-3'
**OCPAna-3aaNtag**	5'-CATCACCATCATCACCACGCAATTACTATCGATTCC-3‘<br>5'-GTGGTGATGATGGTGATGGCTGCTGCCCATGGT-3’

Primer names	Primer sequences
**OCPSyn-NC15aaCtag**	Mutagenesis using pCDF-OCPsynCter<br>5‘AATCAGGCTAATCAGGTGACTCTAAATCCACAAGTTCCATTCACCATTGACTCTGCC-3‘<br>5'AACTTGTGGATTTAGAGTCACCTGATTAGCCTGATTGCTGCTGCCCATGGTATATCTCCT-3'
**OCPSyn-MIX15aaCtag**	5'CGACTAGACAATCCAGAAAAAACTGACATTGAACCACCATTCACCATTGACTCTGCC-3'<br>5'TGGTTCAATGTCAGTTTTTTCTGGATTGTCTAGTCGGCTGCTGCCCATGGTATATCTCCT-3'
**OCPSyn-NC10aaCtag**	5'-AATCAGGCTAATCAGGTGACTCCATTCACCATTGACTCTGCCCGCG-3’<br>5'-AGTCACCTGATTAGCCTGATTGCTGCTGCCCATGGTATATCTCCT-3'
**OCPSyn-NC8aaCtag**	5'-AATCAGGCTAATCAGCCATTCACCATTGACTCTGCCCGCGGA-3’<br>5'-CTGATTAGCCTGATTGCTGCTGCCCATGGTATATCTCCT-3'
**OCPSyn-NC6aaCtag**	5'-CCATTCACCATTGACTCTGCCCGCGGAATT-3‘<br>5'-AGAGTCAATGGTGAATGGAGCCTGATTGCTGCTGCCCATGGTATATCT-3'
**OCPSyn-NC3aaCtag**	5'-CCATTCACCATTGACTCTGCCCGCGGAATT-3‘<br>5'-AGAGTCAATGGTGAATGGGCTGCTGCCCATGGTATATCTCCT-3'
**OCPSyn-C8aaCtag**	5'-AAAAAGAGAAGGGCTCCATTCACCATTGACTCTGCCCGCGGA-3‘<br>5'-AGCCCTTCTCTTTTTGCTGCTGCCCATGGTATATCTCCT-3'

Sup Fig 7: List of synthetic oligonucleotides used to amplify and to clone *ocp* genes and genes involved in carotenoid synthesis.

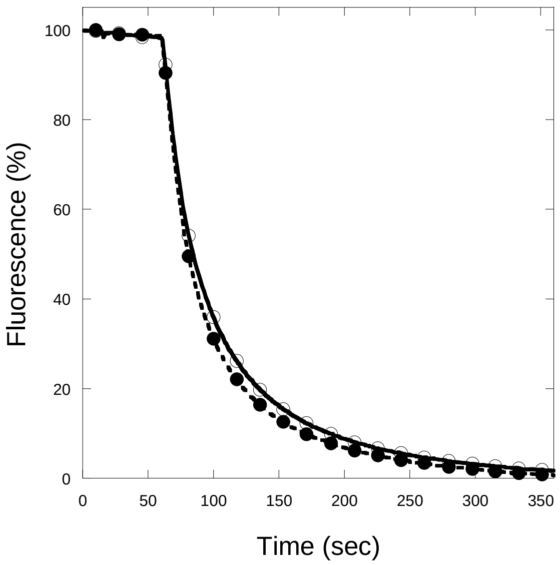

Sup Fig 8: **Apo-protein effect during phycobilisome quenching**

Synechocystis PBS (0.012 μM) were illuminated (30 sec, 900 photons m^{-2} s^{-1} of blue-green light) and then fluorescence quenching was induced by addition of pre-converted *Syn*-3aaNtag-ECN-OCP^{r} (0.48 μM) in the absence (closed symbols) or the presence of 0.48 μM of apo-OCP (open symbols) in 0.5 M phosphate buffer, in 900 photons m^{-2} s^{-1} of blue-green light.

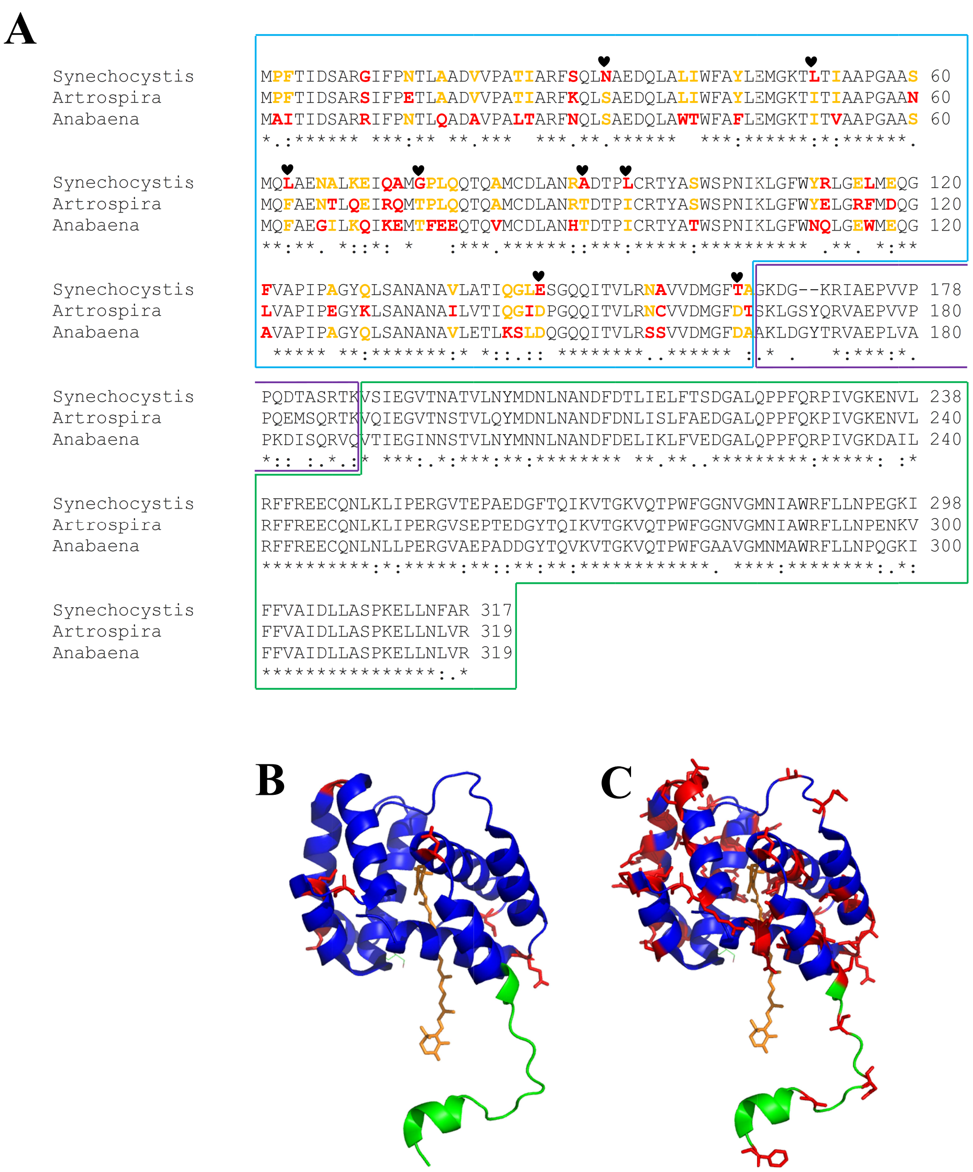

Sup Fig 9: Comparison of different OCPs. (A) Protein sequence alignment of *Synechocystis* PCC 6803, *Arthrospira* PCC 7345 and *Anabaena* PCC 7120 OCPs. In blue, OCP N-terminal domain. In green, OCP C-terminal domain. In purple, OCP loop linking both domains. (♥) corresponds to *Synechocystis* different amino acid compared to *Arthrospira* and *Anabaena*. Non-identical amino-acids in the three sequences of the N-terminal domain are marked in orange and red. (B and C) Structure of the N-terminal domain of *Synechocystis* OCP. The N-terminal arm is marked in green. The carotenoid is in orange. (B) In red, amino acids that are present in *Synechocystis* OCP but not in *Anabaena* and *Arthrospira* OCPs . (C) In red, all non-conserved amino acids in the three OCPs.
